# 

**Published:** 1895-06-01

**Authors:** 


Medical Progress and Hospital Clinics—

Clinical Aspects of Scoliosis - - - - - - - 147

Progress in Medicine.—Nervous Diseases - - - - - 149

Progress in Surgery.—Surgery of the Liver and Gall Bladder - 150

Therapeutic Notes—Chloralose - - - - - - - 151

New Appliances and Things Medical - - - - - 152

Annotations.—The Midwives Bill—Doctor or Spy?—The Birmingham Hospital Saturday Fund - - - - - - 145

The Institutional Workshop:—

Practical Departments.—Reck's Steam Disinfector—Air-Tight Cover - - - - - - - - - - 153

- - - - - - - - - - - - 154

The Story of the Insane from Year to Year - - - 154

Hospital Meetings - - - - - - - - 154

Around the Hospitals - - - - - - - - 155

Construction Notes - - - - - - - - 156

Scraps and Gleanings - - - - - - - - 156

The Book World of Medicine and Science - - - - 157

The Student of Medicine - - - - - - - - 157

### EXTRA SUPPLEMENT.—THE NURSING MIRROR.

News from the Nursing World.—Chelsea Hospital for Women—"The Hospital" Convalescent Fund—A Students' Concert—Nursing in South London—Nottingham—Trouble at Macclesfield—Unanimous, but Unwise—Unpaid Companions—Contrasted Qualifications—A Good Example—Short Items - - - - - - - - lix

Elementary Anatomy and Surgery for Nurses. By W. McAdam Eccles, M.B., M.S., F.R.C.S. - - - - lxi

Workhouse Infirmary Nursing Association - - - lxii

Death in Our Ranks - - - - - - - - lxii

Notes and Queries - - - - - - - - - lxii

Notes from Ireland - - - - - - - - - lxiii

Where to Go - - - - - - - - - - lxiii

Everybody's Opinion - - - - - - - - - lxiv

Small-pox in the Desert—A Nurse's Experience - - lxiv

Presentations - - - - - - - - - - lxiv

